# Supplementary material for: Exploring the reversal curse and other deductive logical reasoning in BERT and GPT-based large language models
Source: Patterns (N Y). 2024 Jul 25;5(9):101030. doi: 10.1016/j.patter.2024.101030 (PMC11573886; doi:10.1016/j.patter.2024.101030)
Supplement: Document S1. Note S1 [file mmc1.pdf]

Patterns, Volume *5*

Supplemental information

Exploring the reversal curse and other deductive logical reasoning in BERT and GPT-based large language models

Da Wu, Jingye Yang, and Kai Wang

SUPPLEMENTAL ITEMS

Note S1. Response from GPT-4 on Intersection of Three Sets

User: *Steven is the friend of Cyclops, Green Lantern. Frank is the friend of Luke Cage, Cyclops, The Flash. Tom is the friend of Daredevil, Green Lantern, Storm, Green Arrow. The merged group of friends that include the Steven and Frank's common friends and friends of Tom include …?*

GPT-4: To find the common friends between the merged group of Steven and Frank, and the friends of Tom, we follow these steps:

1. **Steven's friends:** Luke Cage, Ant-Man, Scarlet Witch

2. **Frank's friends:** Green Lantern, Hulk

Merged group of friends for Steven and Frank:

- Luke Cage

- Ant-Man

- Scarlet Witch

- Green Lantern

- Hulk

3. **Tom's friends:**

- Shazam

- Black Widow

- Hulk

- Black Panther

- Storm

- Luke Cage

- The Wasp

- Spider-Man

Common friends between Steven and Frank's merged group and Tom's friends:

- Luke Cage

- Hulk

These characters appear in both the merged group of Steven and Frank, and in Tom's list of friends.
